# A Health Service Research Study on a Low-Threshold Hearing Screening Program for Childhood Cancer Survivors in Switzerland: Protocol for the HEAR Study

**DOI:** 10.2196/63627

**Published:** 2025-05-21

**Authors:** Philippa Jörger, Carina Nigg, Luzius Mader, Sven Strebel, Martin Kompis, Zuzana Tomášiková, Christina Schindera, Gisela Michel, Nicolas Xavier von der Weid, Marc Ansari, Nicolas Waespe, Claudia Elisabeth Kuehni

**Affiliations:** 1 Institute of Social and Preventive Medicine University of Bern Bern Switzerland; 2 Graduate School for Health Sciences University of Bern Bern Switzerland; 3 Cancer Registry Bern Solothurn University of Bern Bern Switzerland; 4 CANSEARCH Research Platform in Pediatric Oncology and Hematology Department of Pediatrics, Gynecology and Obstetrics University of Geneva Geneva Switzerland; 5 Department of Ear, Nose and Throat Diseases, Head and Neck Surgery Inselspital, University Hospital Bern and University of Bern Bern Switzerland; 6 Childhood Cancer Switzerland Basel Switzerland; 7 Department of Pediatric Hematology and Oncology University Children's Hospital Basel (UKBB) and University of Basel Basel Switzerland; 8 Faculty of Health Sciences and Medicine University of Lucerne Lucerne Switzerland; 9 Division of Pediatric Oncology and Hematology, Department of Women, Child, and Adolescent University Hospital of Geneva and University of Geneva Geneva Switzerland; 10 Division of Pediatric Hematology/Oncology, Department of Pediatrics University Children's Hospital Bern and University of Bern Bern Switzerland

**Keywords:** hearing screening, hearing problems, ototoxicity, platinum compounds, cranial radiotherapy, low-threshold screening, aftercare, childhood cancer survivors, participatory research, long-term follow-up, implementation science

## Abstract

**Background:**

Hearing loss is a common late effect in childhood cancer survivors, caused by ototoxic cancer treatments, such as platinum chemotherapy, cranial radiation with doses of ≥30 Gray, and surgery involving the auditory system. Early recognition of hearing loss as part of follow-up care allows for therapeutic support to mitigate consequences. However, hearing tests are usually only repeated in childhood cancer survivors with abnormal hearing during or right after treatment ends, leaving hearing loss undetected in childhood cancer survivors with late onset or when missed during cancer treatment. Further, general follow-up care attendance may be low after childhood cancer survivors transition to adult care, contributing to missing hearing screening posttherapy. Low attendance may be attributed to childhood cancer survivors finding follow-up care burdensome and time-consuming, lacking awareness of their risk for certain late effects, or the absence of suitable interdisciplinary follow-up clinics. A low-threshold, easily accessible screening program requiring minimal participant effort may address these barriers and improve access to hearing loss screening for childhood cancer survivors.

**Objective:**

The HEAR study aims to develop, conduct, and evaluate the feasibility of a low-threshold, community-based screening program for hearing loss in childhood cancer survivors, using the Reach, Effectiveness, Adoption, Implementation, and Maintenance (RE-AIM) framework, a tool to plan and evaluate health interventions. Within the screening program, participating childhood cancer survivors completed a standardized hearing assessment at a local Swiss hearing aid provider’s shop. This approach provides low-threshold access to detect hearing loss as it is easily and conveniently accessible for everyone.

**Methods:**

Eligible childhood cancer survivors were identified through the Childhood Cancer Registry Switzerland and included those diagnosed with cancer between 1976 and 2019 before 21 years and who were ≥2 years post diagnosis. We invited eligible childhood cancer survivors by post. Participants scheduled a hearing test appointment at a hearing aid shop. They completed a baseline questionnaire before the hearing test, and 2 follow-up questionnaires afterward to assess program feasibility and participant experiences. Semistructured interviews with participants, hearing aid shop staff, and group discussions with health care professionals will provide qualitative insights. The RE-AIM framework will guide the program evaluation using the quantitative and qualitative data collected.

**Results:**

As of February 2025, all participants have been recruited, and all steps of the study up to the group discussions and the RE-AIM evaluation have been completed.

**Conclusions:**

The HEAR study introduces a novel, simple, and low-threshold approach to screening for hearing loss after cancer treatment through hearing aid shops located in the community and close to participants’ homes. This approach has the potential to supplement existing follow-up care programs by reducing the burden of hearing screening for adult childhood cancer survivors and reaching those who might otherwise be lost to follow-up.

**Trial Registration:**

ClinicalTrials.gov NCT06036407; https://clinicaltrials.gov/study/NCT06036407

**International Registered Report Identifier (IRRID):**

DERR1-10.2196/63627

## Introduction

One in 10 survivors of childhood cancer experiences hearing loss [1]. Hearing loss is often caused by ototoxic treatments such as platinum chemotherapy, cranial radiation ≥30 Gray, and surgery involving the auditory system [1-3]. Aminoglycoside antibiotics and loop diuretics, commonly used during acute cancer treatment, are also known to have ototoxic effects [4,5]. Other chemotherapeutics, such as vinca alkaloids, have been associated with hearing loss more recently [6,7]. Undetected hearing loss can affect neurocognitive function, language development, school performance, and overall quality of life of childhood cancer survivors [8-11]. In a previous study, we showed that the incidence of self-reported hearing loss in childhood cancer survivors exposed to platinum chemotherapy and cranial radiation increased up to 63% 15 years after treatment [1]. Similarly, a US study found that the cumulative incidence of hearing loss continued to rise with age in medulloblastoma survivors, emphasizing the potential long-term consequences of ototoxic treatments [12]. Regular hearing tests are key for early detection, allowing timely interventions like hearing aids or speech therapy [13]. The International Guideline Harmonization Group for late effects of childhood cancer recommends hearing screening of childhood cancer survivors after treatment with cisplatin (with or without carboplatin) or cranial radiotherapy ≥30 Gray annually for children <6 years, every other year for children aged 6-12 years, and every 5 years from age 12 years onwards [2]. However, we found that in Switzerland, adherence to audiological monitoring in the clinics was insufficient, with one-third of at-risk childhood cancer survivors lacking posttreatment auditory follow-up screening [14].

In Switzerland, different models exist to monitor late effects once childhood cancer survivors leave pediatric care. Childhood cancer survivors may transition to a general practitioner, seek adult hematology/oncology services, or attend a specialized, interdisciplinary follow-up clinic established in some hospitals [15-17]. However, general follow-up care attendance in adult childhood cancer survivors is only around 40% [17-19]. Even when childhood cancer survivors received audiological monitoring after the end of treatment, there remains a risk that those who no longer attend follow-up care may develop late-onset hearing loss, resulting in undetected cases. Low attendance may be the result of childhood cancer survivors lacking awareness of the risk of late effects and long-term follow-up care options, or experiencing follow-up care as burdensome. Especially young adults may be hesitant to take time off work to revisit medical facilities associated with potentially traumatic memories [18,20,21]. Medical costs may pose an additional barrier.

Low-threshold screening programs could prove useful in addressing these challenges [18]. By “low-threshold screening programs” in this context, we mean programs that minimize barriers to participation as they are intended to be easily accessible, require little time and financial resources, and are integrated into familiar, community-based settings. Such programs aim to provide convenient access to simple, yet high-quality medical tests in nearby facilities. Examples of successful low-threshold programs for the general population include hearing tests in hearing aid shops, influenza vaccinations, blood pressure and blood sugar checks in pharmacies, and walk-in clinics for sexual health [22-24]. To our knowledge, there are no low-threshold programs tailored to screen for late effects in childhood cancer survivors. Offering a low-threshold screening program for adult childhood cancer survivors after transitioning from pediatric care may supplement existing follow-up care options, potentially enhancing overall attendance rates.

The HEAR study aimed to develop and assess the feasibility and effectiveness of a low-threshold screening program for detecting hearing loss in childhood cancer survivors. In this study protocol, we provide an overview of the study design, participant identification, data collection, and program evaluation procedures.

The main objectives of the HEAR study were to (1) develop and pilot a low-threshold community-based screening program for hearing loss among adult childhood cancer survivors in Switzerland, which could supplement existing follow-up care; (2) evaluate the screening program using the Reach, Effectiveness, Adoption, Implementation, and Maintenance (RE-AIM) framework [25,26]; and (3) assess the feasibility of maintaining or expanding the program.

## Methods

### Study Population

**Figure 1:**
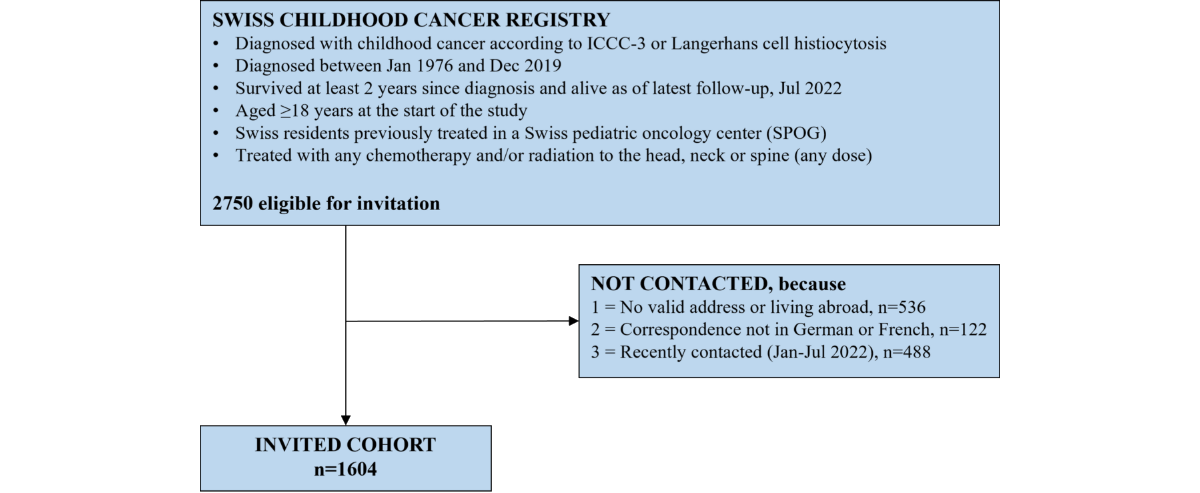
Flowchart illustrating eligibility criteria and total invited cohort of childhood cancer survivors for the HEAR study (N=1604). ICCC-3: International Classification of Childhood Cancer, edition 3.

We identified eligible study participants through the Childhood Cancer Registry Switzerland (ChCR). The ChCR is a national, population-based registry in Switzerland. It includes all individuals diagnosed with leukemia; lymphoma; central nervous system tumor; malignant solid tumor according to the International Classification of Childhood Cancer, third edition; or Langerhans cell histiocytosis before age 21 years since 1976 [27]. Eligible for the HEAR study were childhood cancer survivors registered in the ChCR, who had been diagnosed with childhood cancer between 1976 and 2019, had survived ≥2 years since their diagnosis, were aged ≥18 years at the start of the study, and were at risk for hearing loss (Figure 1). We defined 2 risk groups for hearing loss: The high-risk group included childhood cancer survivors who had been exposed to established ototoxic treatments as defined by the International Guideline Harmonization Group guidelines, namely cisplatin, carboplatin, or cranial radiation ≥30 Gray [28]. The standard-risk group included childhood cancer survivors exposed to other treatments, including any non-platinum-based chemotherapy, radiation to the head with <30 Gray, or neck or spine with any radiation dose. Although these treatments are not explicitly defined as ototoxic, previous studies suggest that these treatments still carry a higher risk of developing hearing loss compared with siblings, likely due to the use of other suspected ototoxic agents such as aminoglycoside antibiotics, loop diuretics, or cerebral spinal fluid shunts [1,29].

We excluded childhood cancer survivors who had only received radiotherapy to areas other than the head, neck, or spine due to a low risk of developing hearing loss; those who had been treated with surgery only since information regarding the exact surgery location was not readily available in the ChCR; those who did not speak German or French due to limited resources for study material translation; those who had been contacted in the last 6 months for another study (January-June 2022) to avoid overburdening, and those for whom we could not retrieve a valid address or who lived abroad.

### Study Design and Study Procedures

**Figure 2:**
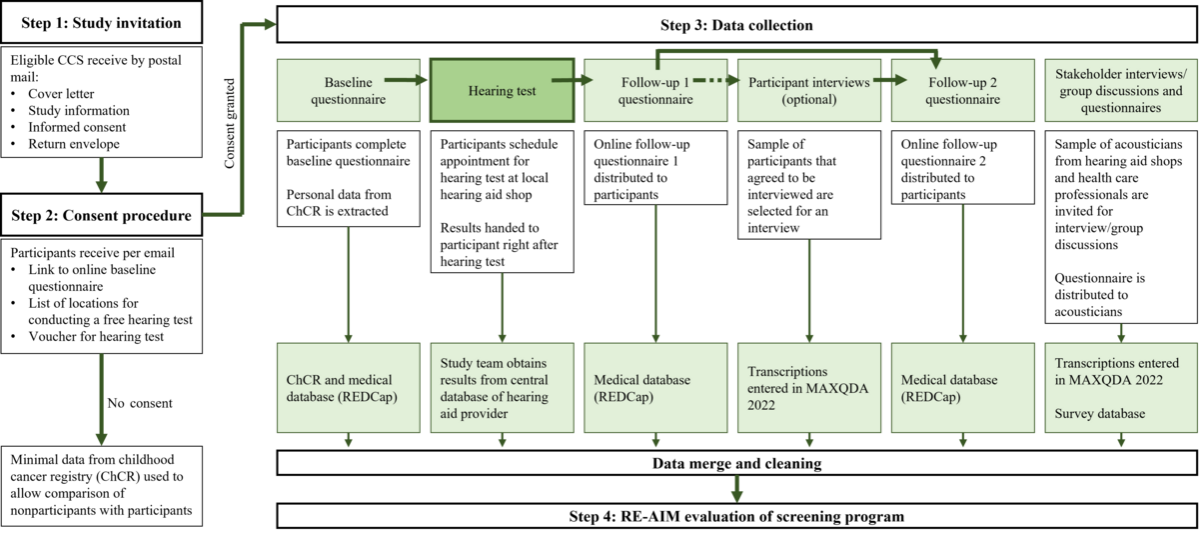
Schematic chart of study design and study procedures of the HEAR study. CCS: childhood cancer survivors; ChCR: Childhood Cancer Registry; REDCap: Research Electronic Data Capture.

Figure 2 illustrates the study procedures. We describe the process in detail in the following.

#### Step 1: Study Invitation

Eligible childhood cancer survivors identified from the ChCR received a cover letter, study information, informed consent, and a prepaid return envelope (Figure 2). We provided contact details (study phone hotline, and email) for questions or comments. Nonresponders received up to 2 written reminders after 3 to 9 weeks based on response patterns and seasonality (eg, avoiding holidays).

#### Step 2: Consent Procedure

After participants returned the consent form, we invited them by mail to attend a free hearing test at a local hearing aid shop, providing them with a voucher for the hearing test, a list of available shops across Switzerland together with contact information (email and telephone), and a link to complete a baseline questionnaire via a secure web-based platform.

#### Step 3: Data Collection

##### Baseline Questionnaire

In the baseline questionnaire, we asked for baseline information about participants’ hearing. We sent up to 3 reminder emails, followed by a final reminder letter by regular mail if needed, addressing potential email errors. For details about the baseline questionnaire, see section “Data Collection: Quantitative.”

##### Hearing Test

Participants themselves scheduled an appointment at a hearing aid shop of their choice. The hearing aid shops belong to a hearing aid provider with branches located throughout Switzerland. At the hearing aid shop, a certified acoustician first performed a video otoscopy to examine the condition of the external auditory canal and tympanic membrane. The acoustician then performed a bilateral pure tone audiometry from 125 Hz to 8000 Hz and, for participants with hearing loss >25 decibels, bone conduction from 250 Hz to 4000 Hz. The acoustician handed out and explained the hearing test results to the participants during the same visit. If results were outside the normal range, the acoustician advised participants to contact their family doctor; an ear, nose, and throat specialist; or an audiologist. We offered participants without a medical contact person to contact the study team for assistance in finding appropriate counseling. The hearing aid provider collected the coded audiogram data and digital otoscopy images from all hearing tests on their central database and sent them to us in an encrypted digital form after all participants had completed the hearing tests by the end of July 2023. Data from all locations were pooled for analysis.

##### Follow-Up 1 and 2 Questionnaires

After the hearing test, we sent participants 2 follow-up questionnaires by mail, a follow-up 1 questionnaire as soon as we got informed that a participant completed a hearing test, and a follow-up 2 questionnaire between 7 and 12 months after the hearing test. For follow-up questionnaires, we sent up to 2 reminder emails. For details about follow-up questionnaires, see section “Data Collection: Quantitative.”

##### Participant Interviews

We also invited a subgroup of participants who expressed interest and completed the follow-up questionnaire 1 for personal interviews. We contacted potential interviewees by email, informing them in detail about the interview process and objectives. Interviewees could choose to participate either via video call or in person. Before the interview, interviewees signed an informed consent form. More details about the interviews can be found in the section “Data Collection: Qualitative.”

##### Stakeholder Interviews/Group Discussions and Questionnaires

After the last participants had visited the hearing aid shops, we interviewed a sample of acousticians who had carried out the hearing tests, and the hearing aid providers’ contact person. With the interview outputs, we developed a questionnaire for acousticians to gather insights from a larger sample. We emailed acousticians a link to an anonymous web-based questionnaire.

We intend to organize group discussions with health care professionals to get input on the screening program from all stakeholders involved.

#### Step 4: RE-AIM Evaluation of Screening Program

The generated data will be used to evaluate the screening program using the RE-AIM framework [25,26]. We explain the RE-AIM evaluation in more detail in the section “RE-AIM Evaluation Framework.”

### Ethics Approval

The Ethics Committee of the Canton of Bern, Switzerland, provided ethical approval for this study (KEK-BE 2021-01624). The study was registered at ClinicalTrials.gov (identifier: NCT06036407). Participants provided informed written consent for study participation and permission to obtain the hearing test results from the hearing aid shops to use for research. If any patient declined to participate, their data were not collected, and they could decide to exit the study at any time point. All generated data excluded names or other identifying information. To protect the participants’ privacy, the hearing aid shop employees were not informed about the medical history of the participants and were not allowed to ask participants about it during the test. Employees were only informed that participants had been exposed to some treatment in their childhood or adolescence, potentially leading to hearing loss. Further, hearing aid shop employees were not allowed to advertise any products to participants. Participants were not compensated for their efforts. This study protocol has been prepared in accordance with the Standards for Reporting Implementation Studies (StaRI) guidelines [30]. As this is a study protocol, items related to results and findings have not been addressed.

### Patient and Public Involvement

For this participatory research project, we set up a stakeholder advisory group with 3 childhood cancer survivors, one of them working for a patient advocate organization, 5 health care professionals, and 2 representatives of the hearing aid provider. We consulted the group regarding the study design and communication with study participants, with a focus on ethical questions, information material for participants, and questionnaire/interview guide development. The advisory group members met repeatedly with the study team to discuss the ongoing study. All study participants had the opportunity to provide feedback in the follow-up questionnaires. The study team regularly reviewed feedback from questionnaires and incorporated it into the ongoing study whenever feasible.

### RE-AIM Evaluation Framework

#### Overview

To assess the feasibility and effectiveness of the screening program, we plan to use the RE-AIM framework, a well-established tool for assessing the public health impact of health interventions [25]. The domains guiding the evaluation are Reach, Effectiveness, Adoption, Implementation, and Maintenance. We will integrate both qualitative and quantitative data sources to evaluate each domain. In the following section, we provide an overview of how we plan to apply the RE-AIM framework to evaluate the screening program.

#### Reach

Reach measures the extent to which the target population participates in the program. We aim to assess the number and proportion of the invited childhood cancer survivors who accepted the offer of a nearby hearing test and compare their characteristics with nonparticipants, including sex, age at survey and diagnosis, type of diagnosis, risk group, and language region. We intend to use data on clinical information from the ChCR and additional information from questionnaires to characterize participants. We plan to identify motives for participation using questionnaires and personal interviews with participants.

#### Effectiveness

Effectiveness focuses on evaluating the program’s immediate and long-term impact (positive and negative) and whether the program achieves its objectives. Using audiogram results and self-reported information from questionnaires, we plan to analyze the number and proportion of participants with (newly) detected hearing loss, the proportion of those who contacted a physician for further evaluation, and the number of those acquiring a hearing aid after the screening program. Upon comparison to other populations, we will consider age-related differences in hearing loss prevalence. We plan to assess possible other effects of the program through questionnaires and interviews with participants.

#### Adoption

Adoption assesses the stakeholder’s willingness to adopt and implement the program. Through questionnaires and interviews with stakeholders (hearing aid shop employees and health care professionals) and childhood cancer survivors, we plan to explore experiences and satisfaction with the screening program. We intend to assess the number and distribution of shops visited during the study using information from the hearing aid provider.

#### Implementation

Implementation evaluates the feasibility of implementing the program within real-world contexts, assessing the extent to which the program was delivered consistently and as intended. It also includes an estimation of required resources. We plan to study compliance with the program as well as facilitators and barriers to program implementation through interviews and questionnaires with stakeholders and childhood cancer survivors. We intend to estimate required resources based on information gathered through discussions with the hearing aid provider’s contact person and health care providers.

#### Maintenance

Maintenance investigates the possibility of continuing the program beyond the research project for long-term sustainability. We plan to use data from questionnaires and interviews to get stakeholders’ and childhood cancer survivors’ insights and to identify necessary requirements and resources for the program’s continuation.

### Data Collection: Quantitative

#### Clinical Data, Otoscopies, and Audiograms

The ChCR provided data on diagnosis; relapse; age at cancer diagnosis; year of cancer diagnosis; therapy type (chemotherapy, radiotherapy, surgery, or hematopoietic stem cell transplantation); cumulative doses of platinum-based chemotherapy (where available); and documented radiation dose (gray) of the field involving the head, neck, and spine. For eligible childhood cancer survivors who did not consent or respond to participate, the ChCR provided minimal data (sex, year of birth, year of diagnosis, cancer diagnosis, and therapy information). We will use this minimal data to compare participants to nonparticipants.

All hearing aid shops followed the same protocol and designated acousticians responsible for the study. The audiometries were carried out by standardized regulations (ISO [International Organization for Standardization] 9001) using audiometers and audiogram software from Natus Medical Incorporated [31]. The measurement equipment (audiogram and hearing test cabin) is subject to the Ordinance of the Federal Department of Justice and Police on Audiometric Measuring Equipment (Audiometry Ordinance) [32].

#### Study Participants and Acoustician Questionnaires

The information gathered in the interviews and questionnaires should help shape the program so that it can be used as an integral part of care for childhood cancer survivors. The questions within the questionnaires consisted of a combination of multiple-choice and open-ended questionnaire items, with the majority being multiple-choice items that can be statistically analyzed. The open-ended responses provide additional context but are not the primary source of qualitative analysis. For qualitative analysis, the primary sources are individual interviews and group discussions (see section “Data Collection: Qualitative”). Table 1 summarizes the study participants and the acoustician questionnaire’s content (baseline, follow-up 1, follow-up 2, and acoustician).

**Table 1:** Content summary of questionnaires sent to participants (baseline, follow-up 1, follow-up 2 questionnaires) and acousticians (acoustician questionnaires).

Questionnaire	Content summary
Baseline questionnaire	Personal, demographic, and socioeconomic data Reasons for participation and expectations Follow-up care attendance and perceived late effects of cancer and cancer treatments Previous hearing tests and hearing monitoring Hearing problems during childhood and at time of survey Risk factors for hearing problems Tinnitus
Follow-up 1 questionnaire	Hearing test experience Appointment scheduling, travel duration, transportation means, testing duration, and problems during shop visit Feelings during/after the testing Understanding of results and information on how to proceed Choice of location of hearing aid shop Comparison to previous hearing tests Opinions on hearing test opportunity Interview participation
Follow-up 2 questionnaire	Actions taken after receiving the results Intentions to attend future hearing tests Facilitators and barriers for hearing tests Advantages/disadvantages of different hearing test opportunities (ENT^a^ specialist, general practitioner, and hospital) Opinion on general follow-up care General study feedback
Acoustician questionnaire	General experiences with study participants Problems encountered during the hearing test Information provided to participants Differences between study participants and usual customers Opinions on the program and on the continuation of it Minimal personal demographics (age, gender, and training)

^a^ENT: ear, nose, and throat specialist.

We collected and managed questionnaire data using the REDCap (Research Electronic Data Capture; version 13.7.5; Vanderbilt University) tool hosted at the University of Bern, a secure, web-based software platform to support data capture for research studies [33,34].

### Data Collection: Qualitative

#### Overview

Interviews with study participants and stakeholders facilitated in-depth insights into different perspectives, experiences, and opinions on the screening program, allowing a comprehensive program evaluation.

#### Interviews

We developed semistructured interview guides with input from the stakeholder advisory group and researchers with expertise in qualitative research. The initial guide version served as a fundamental question catalog that we iteratively refined based on feedback and insights from the first few interviews. Changes improved question clarity, sequence, and wording. An overview of the topics discussed in the respective interviewed groups is provided in Table 2.

**Table 2:** Interview guide content summary for participants and stakeholders (acousticians from the hearing aid shops and health care professionals).

Questionnaire	Content summary
Participant interview	Experiences at the hearing test Opinions on the screening program Advantages/disadvantages of conducting a hearing test in a hearing aid shop compared with more clinical settings (ENT^a^, general practitioner, multidisciplinary follow-up clinic, and hospital) Preferences when conducting a hearing test Experiences with and opinions on general follow-up care Feedback on the study
Stakeholder interviews/group discussions	Experiences with and opinions on the program Challenges or difficulties encountered/anticipated For acousticians from hearing aid shops: Information provided to participants Differences between usual customers and study participants For health care professionals: Opinion on this community-based screening program Integration into existing follow-up care Opinions on feasibility for continuation beyond the research project Necessary requirements and resources for the program’s continuation beyond research project

^a^ENT: ear, nose, and throat specialist.

We selected study participants using purposive sampling to ensure a balanced representation across sex, age, risk groups, education level, and language regions (German and French).

For interviews with acousticians, the hearing aid provider’s contact person suggested 5 acousticians. Additionally, we invited the hearing aid provider’s contact person for an interview.

#### Group Discussions

For the group discussions, we plan to invite health care professionals from the field of pediatric oncology, follow-up care, and audiology to gather their insights regarding the potential continuation of this screening program to supplement follow-up care for adult childhood cancer survivors in Switzerland. At this stage, we are planning smaller group discussions (n=2-3 participants per group) with pediatric oncologists; ear, nose, and throat specialist; and general practitioners to review the study findings, share experiences with the program, and explore its feasibility, as well as potential strategies for implementation. Depending on the outcomes of these group discussions, we will plan a larger focus group, with the number of people and composition of participants depending on the outcomes of the small group discussions.

### Data Analysis: Quantitative

We will conduct quantitative data analysis using STATA (version 16; StataCorp) or RStudio (R Core Team). We plan to use descriptive statistics to assess baseline characteristics of the study population, together with chi-square tests, *t* tests, and analysis of variance for group comparisons.

### Data Analysis: Qualitative

We recorded all interviews. A research assistant translated all French interviews into German. We transcribed the recordings verbatim into Microsoft Word before importing them into MAXQDA 2022 (VERBI Software) for detailed analysis. We verified all transcriptions against the audio recordings.

We will use thematic analysis as described by Braun and Clarke [35,36] as a guiding framework for data analysis of interviews and group discussions. Thematic analysis will allow us to systematically identify, analyze, and interpret themes emerging within the data.

As this protocol focuses on describing the study design and methodology, a detailed discussion of the data analysis processes (quantitative and qualitative) will be presented in future publications alongside the study results.

### Dissemination

We intend to disseminate research findings via national and international conferences and publications in peer-reviewed journals. We plan to share the study results with the participants in the form of newsletters for a lay audience.

## Results

We invited 1604 eligible childhood cancer survivors for study participation and recruited between July 2022 and January 2023. We invited participants to visit hearing aid shops for hearing tests and complete baseline questionnaires until July 2023 and follow-up questionnaires until July 2024. Interviews were scheduled between October 2022 and April 2023 for study participants, and between November 2023 and January 2024 for hearing aid shop employees. Group discussions with health care professionals are still in the planning stage; they are planned for early spring 2025. Analysis of quantitative and qualitative results for evaluation of the program using RE-AIM is ongoing.

## Discussion

### Summary

The HEAR study develops, conducts, and evaluates a new, low-threshold, community-based screening program for detecting hearing loss in adult childhood cancer survivors. Given the increased risk of hearing impairment from ototoxic treatments, the study aims to provide convenient access to high-quality hearing tests at local hearing aid shops, facilitating audiology follow-up care for adult childhood cancer survivors at risk [37,38]. The initiative could be used to supplement existing follow-up care and may serve as a model that could be extended to community-based screening programs for other late effects in adult childhood cancer survivors. The study protocol outlines the design, participant identification, data collection of the study, and program evaluation procedures.

### Comparable Screening Programs for Childhood Cancer Survivors

Until now, no other comparable screening program for adult childhood cancer survivors exists to our knowledge. The presented screening program describes a decentralized option for follow-up care in the community outside the conventional medical facility setting. This novel initiative aimed to provide a more approachable option for adult childhood cancer survivors to undergo recommended screenings, broadening the range of follow-up options available to them. The approach taken in this study contributes to the empowerment of childhood cancer survivors, since the low-threshold and low-cost approach gives survivors the choice to have their hearing tested and to decide how they handle the results.

### Scaling-Up Potential and Future Vision

Given the widespread presence of hearing aid shops in many parts of the Western world, this program could serve as a model for reaching childhood cancer survivors who may otherwise be lost to follow-up. The approach bears a large potential for scalability. Beyond audiological follow-up care, a similar approach could be extended to address other potential late effects of childhood cancer, such as screening for treatment-induced cataracts [39] in opticians’ stores, or diabetes [40], high blood pressure, and hypercholesterinemia [41] in pharmacies.

### Study Limitations

Although we aimed for a low-threshold screening approach, the study procedures requested by the ethics committee were the same as for conventional clinical studies, involving detailed information and legal consent forms. Being cognitively overwhelmed by trial information and confused by scientific and legal terminology in the consent form has been found to be a significant barrier to trial participation [42]. These procedures may have counteracted the intended low-threshold approach and may have mitigated the representativeness of the childhood cancer survivors taking part in the program.

The acousticians conducting the audiological evaluations lacked detailed knowledge about the medical history of the participants. This may have hindered their ability to perform a comprehensive audiological evaluation and client service. The results of the questionnaires and interviews with acousticians will allow us to evaluate the impact of this limitation.

There may have been a selection bias in study participation. Those who participated may have been interested in their hearing health, maybe due to existing problems. Childhood cancer survivors uninterested in the topic may have participated less. Further, despite aiming for a low-threshold approach, childhood cancer survivors with limited mobility may have been unable to participate in the screening program. Finally, the program cannot ensure that childhood cancer survivors with identified hearing problems are followed up appropriately in a medical setting, since it is the decision and responsibility of the childhood cancer survivors to organize a medical follow-up visit together with their physician.

### Conclusions

The decentralized, low-threshold approach for follow-up care offers an alternative to centralized health care settings and allows adult childhood cancer survivors to access follow-up hearing assessments close to their homes. This approach saves time, reduces potential emotional stress associated with clinic visits, and minimizes health care costs by directing only those with abnormal screening outcomes toward further medical evaluation.

As such, this low-threshold hearing loss screening program for childhood cancer survivors may be a step toward enhancing reach and accessibility for hearing loss screening. If positively evaluated, this could be a valuable opportunity to supplement existing follow-up care, lowering the burden of hearing screening for adult childhood cancer survivors and maybe reaching those who would be otherwise lost to follow-up.

## Abbreviations

ChCR: Childhood Cancer Registry
ISO: International Organization for Standardization
RE-AIM: Reach, Effectiveness, Adoption, Implementation, and Maintenance
REDCap: Research Electronic Data Capture
StaRI: Standards for Reporting Implementation Studies

## Data Availability

The datasets generated or analyzed during this study are not publicly available due to ethical restrictions, but are available from the corresponding author on reasonable request.
